# Expression of *hes*, *iha*, and *tpsA* codified in locus of adhesion and autoaggregation and their involvement in the capability of shiga toxin-producing *Escherichia coli* strains to adhere to epithelial cells

**DOI:** 10.1186/s13104-023-06433-9

**Published:** 2023-08-07

**Authors:** Rocío Colello, M. Victoria Vélez, M. Victoria Nieto Farias, Marcelo Rodríguez, David Montero, Roberto Vidal, Analía I. Etcheverría, Nora Lía Padola

**Affiliations:** 1 Facultad de Ciencias Veterinarias, Universidad Nacional del Centro de la Provincia de Buenos Aires (UNCPBA), CISAPA, Tandil, Buenos Aires 7000 Argentina; 2 Centro de Investigación Veterinaria de Tandil (CIVETAN), UNCPBA- CICPBA- CONICET, Tandil, 7000 Argentina; 3 Facultad de Ciencias Veterinarias, Universidad Nacional del Centro de la Provincia de Buenos Aires (UNCPBA), SAMP, Tandil, Buenos Aires 7000 Argentina; 4 Departamento de Microbiología, Facultad de Ciencias Biológicas, Universidad de Concepción, Concepción, 4030555 Chile; 5 Centro Integrativo de Biología y Química Aplicada (CIBQA), Universidad Bernardo O’Higgins, Santiago, 8320000 Chile; 6 Programa de Microbiología y Micología, Instituto de Ciencias Biomédicas, Facultad de Medicina, Universidad de Chile, Santiago, 8320000 Chile

**Keywords:** STEC, LAA, Adherence capability

## Abstract

**Objectives:**

Shiga toxin-producing *Escherichia coli* strains LAA-positive are important cause of human infection. The capability to adhere to epithelial cells is a key virulence trait, and genes codified in LAA pathogenicity island could be involved in the adhesion during the pathogenesis of LAA-positive STEC strains. Thus, our objectives were to compare *hes*-negative and *hes*-positive STEC strains in their adherence capability to epithelial cells (HEp-2) and to evaluate the expression levels of the *hes*, *iha*, and *tpsA* in the bacteria adhered and non-adhered to HEp-2 cells. These genes are encoded in LAA, and are virulence factors that participate in adhesion and autoaggregation.

**Results:**

We could not observe differences between the adhesion of strains but also in the expression level of of *hes*, *iha*, and *tpsA*. Genes encoded in LAA contribute to the adhesion phenotype though the expression of STEC adhesins is a coordinated event that depends not only the strain but also on the environment as well as its genetic background. Therefore, the results of this study suggest that LAA ,the most prevalent PAI among LEE-negative STEC strains, plays a role in pathogenesis.

**Supplementary Information:**

The online version contains supplementary material available at 10.1186/s13104-023-06433-9.

## Introduction

Shiga toxin-producing *Escherichia coli* (STEC) are important pathogens responsible for foodborne diseases. The most virulent STEC strains isolated from human infections belong to the O157:H7 serotype, but other serotypes can cause several diseases [1, 2]. These pathogens are classified by the presence or absence of the Locus of Enterocyte Effacement (LEE) [3] LEE-positive strains can colonize the intestinal mucosa causing the attaching and effacing (A/E) lesion on the intestinal epithelium [3]. Nevertheless, the presence of LEE is not crucial for the pathogenesis of all the STEC strains since some LEE-negative, such as O113:H21 and O91:H21 have been associated with severe disease in humans [4, 5]. Despite the frequent isolation of STEC LEE-negative, the virulence potential and genetic profiles of clinical STEC isolates remain uncharacterized [5]. STEC LEE-negative could possess alternative virulence factors for adherence. Different pathogenicity islands (PAI) have been reported exclusively in LEE-negative STEC strains, such as Locus of Proteolysis Activity [6] and Locus of Adhesion and Autoaggregation (LAA), harboring diverse arrays of virulence factors [7].

LAA is a four-module structure: module I (*hes*), module II (*iha* and *lesP*), module III (*pagC* and *tpsA*), and module IV (*agn43*) [8, 9]. Montero, et al. [8] have reported the complete sequence of this PAI, in which other virulence factors participated in adhesion and autoaggregation. Hes, is a new member of the Heat-resistant agglutinin family (Hra Family), and this protein was named **He**magglutinin from **S**higa toxin-producing *E. coli*, it is a virulence factor that participates in several phenotypes associated with colonization, including adhesion and autoaggregation [8]. Other virulence factors participating in adhesion and autoaggregation are also encoded in LAA, such as Iha an adherence-conferring protein distributed among LEE-negative and LEE-positive STEC strains [10]. TPS systems are TpsA for translocated proteins to the bacterial surface and TpsB for the transporter proteins. TPS systems participate in diverse virulence phenotypes such as adhesion, invasion, and autoaggregation [11, 12].

In light of these observations, our group was investigating genomic analysis, and PCR assays detected *hes*, *iha*, and *tpsA* in a high percentage of LEE-negative STEC strains from different origins and harboring diverse virulence repertoires [10, 13]. However, their expression levels have not been evaluated yet. The capability of bacteria to adhere and cause infection is associated with the expression of specific outer membrane proteins [14]. STEC genome plasticity provides the pathogen a great potential for genome expansion and niche adaptation [15, 16].

Genes codified in LAA could be involved in the adhesion during the pathogenesis of LAA-positive STEC strains, and little is known about the specific virulence factors that contribute to these pathogeneses. Thus, our objectives were to compare *hes*-negative and *hes*-positive STEC strains in their adherence capability to HEp-2 cells and to evaluate the expression levels of the *hes*, *iha*, and *tpsA* genes in the bacteria adhered and non-adhered to HEp-2 cells.

## Materials and methods

### Strains

A total of twenty O91 STEC were analyzed. All isolates are from the Immunochemistry and Biotechnology Laboratory (FCV-UNCPBA, Argentina) collection. The strains were previously isolated from cattle in Argentina and analyzed for the presence of genes encoding for Stx1/2 and their genetic diversity (Table 1) [7, 9, 17–20]. Seventeen O91 STEC were positive for *hes* (LAA complete), and three O91 STEC were negative for *hes* (LAA incomplete) [9]. *E. coli* HB101 was used as a negative control. Also, the mutant of the O91 STEC strain (VO 7-4-4) generated by the deletion of *hes* (O91Δ*hes*) and *E. coli* HB101 transformed with pVB1_*hes* [8] were included.

**Table 1:** Strain ID, virulence profile, *hes* presence, and CFU/ml

Strains	Origin	Virulence profile	*hes*	CFU/ml	Reference
***E. coli*** **HB101**			-	2.000	[8]
**FO130**	Feedlot Cattle	*stx2, ehxA, saa*, LAA	+	19.250	[9, 18–20]
**VO 92-2-1**	Dairy Cattle	*stx2, ehxA, saa, iha, lesp, agn43*	-	30.250	[9, 19, 20]
**VO 14-4-2**	Dairy Cattle	*stx2, ehxA, saa*, LAA	+	44.750	[9, 19, 20]
***E. coli*** **HB101pvb1_*hes***	Strain transformed		+	51.750	[8]
**VO 8-2-4**	Dairy Cattle	*stx2, ehxA, saa*, LAA	+	53.750	[9, 19, 20]
**O91∆** ***hes***	Mutant	*stx2, ehxA, saa, iha, lesp, agn43*	-	55.500	
**VO 70-2-4**	Dairy Cattle	*stx2, ehxA, saa*, LAA	+	58.750	[9, 19, 20]
**TRN5.1.1**	Growling Calf	*stx2, ehxA, saa*, LAA	+	60.500	[9, 19, 20]
**VO 67-1-3**	Dairy Cattle	*stx2, ehxA, saa*, LAA	+	64.250	[9, 19, 20]
**VO 10-1-4**	Dairy Cattle	*stx2, ehxA, saa*, LAA	+	94.000	[9, 19, 20]
**VO 59-1-1**	Dairy Cattle	*stx2, ehxA, saa*, LAA	+	112.333	[9, 19, 20]
**VO 59-3-2**	Dairy Cattle	*stx2, ehxA, saa*, LAA	+	116.250	[9, 19, 20]
**VO 69-3-2**	Dairy Cattle	*stx2, ehxA, saa*, LAA	+	137.667	[9, 19, 20]
**VO 87-2-1**	Dairy Cattle	*stx2, ehxA, saa*, LAA	+	142.000	[9, 19, 20]
**TR 47-1-1**	Growing Calf	*stx2, ehxA, saa, iha, lesp*	-	172.500	[9, 19, 20]
**TR 15-1-5**	Growing Calf	*stx2, ehxA, saa, iha, agn43, cah*	-	192.500	[9, 19, 20]
**VO 42-2-2**	Dairy Cattle	*stx2, ehxA, saa*, LAA	+	192.500	[9, 19, 20]
**FO135**	Feedlot Cattle	*stx2, ehxA, saa*, LAA	+	200.000	[9, 18, 19]
**VO 59-5-1**	Dairy Cattle	*stx2, ehxA*	+	267.500	[9, 19, 20]
**AP16.1**	Grazing Cattle	*stx2, ehxA, saa*, LAA	+	310.000	[9, 17, 19]
**VO 7-4-4**	Dairy Cattle	*stx2, ehxA, saa*, LAA	+	335.000	[9, 19]

### Culture and inoculation to HEp-2 cells

The cell line was kindly provided by INTA Castelar, Buenos Aires, Argentina. HEp-2 cells were cultured in Minimal Essential Medium added with 10% of fetal calf serum (Natacor®) at 37 °C with 5% CO_2_. The cells were seeded in 24 well plates. The supernatant was discarded, and the plates were washed three times with phosphate-buffered saline (PBS). Then, 900 µl of fresh medium was added to each well. To inoculate the cells, each O91 strain was cultured in Luria Bertani broth at 37ºC for 18 h with shaking. One hundred ml of a culture adjusted by OD_600_ to a concentration of 10^6^ CFU/mL was added to each well. Plates were incubated at 37ºC for 3 h. The monolayer was washed three times with PBS, and 100 µL per well of Trypsin-EDTA was added to recover HEp-2 cells with adhered bacteria. The cells disattached were recovered, and several dilutions of the cell suspensions were seeded onto MacConkey agar plates to quantify colonies corresponding to STEC. The experiments were performed in triplicate [21].

### RNA extraction

The total RNA was extracted from three LAA-positive O91 STEC strains (VO 7-4-4, AP16.1, and TRN 5.1.1) and the *E. coli* HB101 pVB1_*hes* strain. The expression levels of *hes*, *iha*, and *tpsA* were evaluated in adhered and non-adhered in each strain selected from the HEp-2 cell assay. Briefly, the strains non-adhered were recovered from the supernatant after incubation with HEp-2 cells, and then the total RNA was extracted. As detailed above, the strains that adhered to HEp-2 cells were recovered when washing after adding Trypsin. The RNA of strains was extracted using a TRIzol reagent. Two separate experiments with at least three replicates each were conducted.

The treatment with DNase I was made to reduce gDNA contamination before reverse transcription; for this, 1 µg of RNA was incubated with 20 U of DNase I for 1 h at 37 °C followed by 12 min at 72 °C for inactivation. Afterward, cDNA was synthesized by using the High-Capacity cDNA Reverse Transcription Kit. RNA concentration and integrity were evaluated using a Spectrophotometer and electrophoresis, respectively.

### Primer’s design

The primers used to detect *iha* and *tpsA* were described by Colello, et al. [9]. For the *hes*, the strains were previously characterized by PCR with specific primers [8]. The *hes*_RT primers were designed using the Primer3plus software (https://www.primer3plus.com/) (Table 2).

**Table 2:** Primers used and PCR amplicons sizes

Primer	Sequence (5´-3’)	Size (bp)	Reference
*hes*_for	AGGTCATCACGCCAGTAACC	113	This study
*hes*_rev	CAGTTCAGTATTCCGGTTCG		
*iha*_for	TTTCAGCCAGCAGCATGGCA	172	[9]
*iha*_rev	ACATCCACACCCTCCACAGC		
*tpsA*_for	CACCCGTACCGTGGAAGAAACC	174	[9]
*tpsA*_rev	TCGCCACTGACACTGACATTTTCC		

### Quantitative real-time PCR analysis

The reactions consisted of 20 µL: 4 µl of 1/5 diluted cDNA, 10 µl of 2X SYBR Green master mix, and 300 nM of each primer. A no-template control was included in each run to assess for reagent contamination. The PCR was programmed for 40 cycles of 95 °C for 15 s followed by 60 °C for 1 min using the OneStep Plus Real-Time PCR System. The relative quantification was performed for the *hes*, *iha*, and *tpsA* to evaluate the expression of each strain adhered and non-adhered to epithelial cells. For each gene, the expression values were analyzed individually and independently, comparing adhered strains with their respective non-adhered strains. The data for the expression assays were detected in three separate experiments, and negative controls were included in each plate.

The reference gen *tufA* was used as the housekeeping [22]. The expression level of each gene was calculated by the relative fold change by the ΔΔCt threshold cycle (CT) method using the efficiency corresponding to each gene, which was obtained from the relative standard curves and determined by using the OneStep Plus Real-Time PCR System (Figure S1, S2, S3, and S4) [23]. Data sets were log10-transformed, and if the fold change is positive, the gene is upregulated; if the fold change is negative, it is downregulated [24].

### Statistical analysis

Separate experiments were conducted to compare *hes*-negative and *hes*-positive STEC strains in their adherence capability to HEp-2, and a general linear model was fitted. A p-value of less than 0.05 was considered significant. The analyzes were performed with the R 3.6.3, lme4, and Multcomp programs [25, 26].

## Results and discussion

**Fig. 1:**
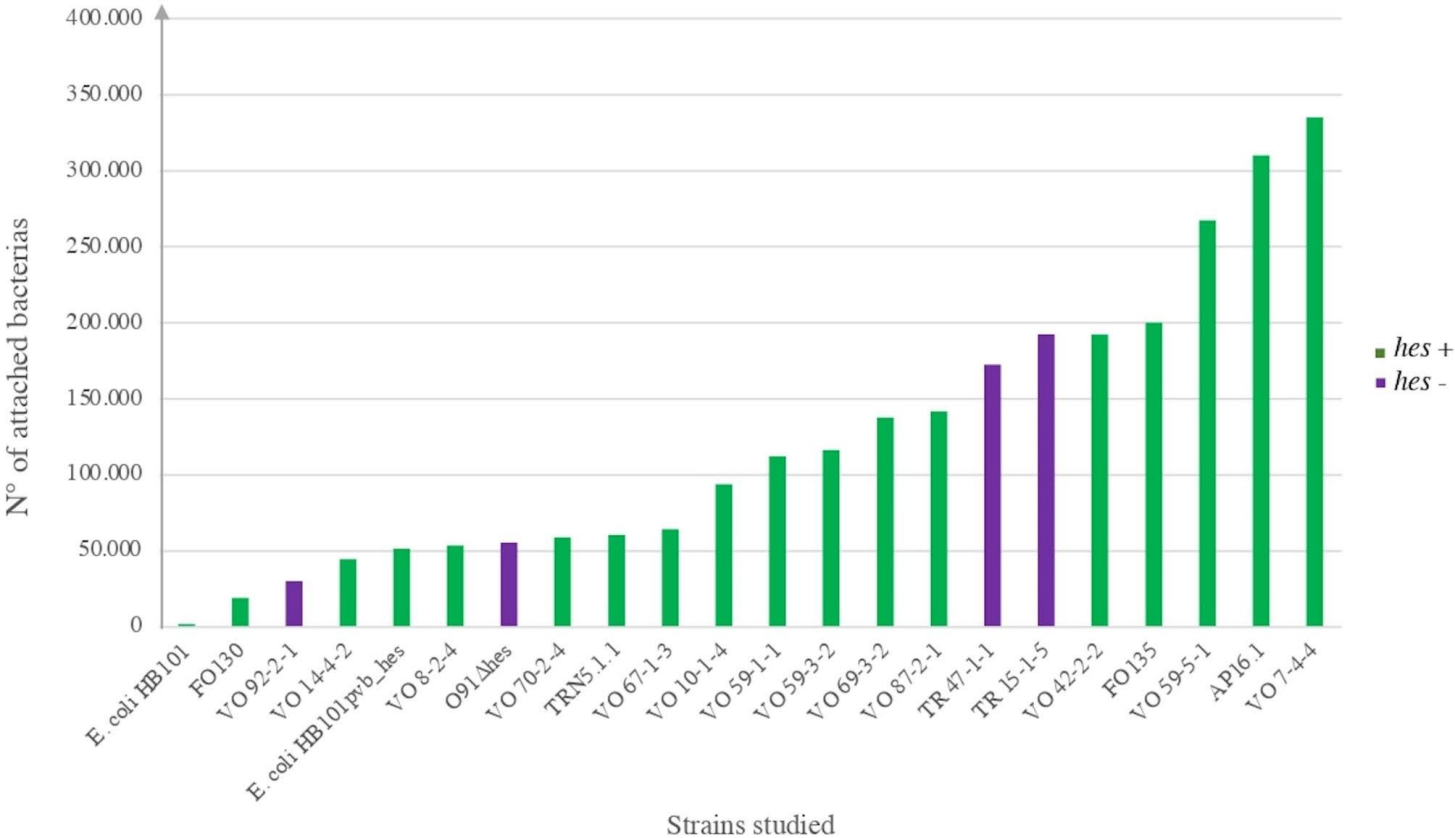
Adherence to HEp-2 by the O91 STEC strains The adhered strains are expressed as the number of CFU per milliliter. O91Δ*hes* STEC and *E. coli* HB101 pVB1_*hes* were used to compare their adherence with the adherence of the O91 STEC strains and the capability to adhere to HEp-2 cell was variable. *Green bars are *hes* positive strains and violet bars are *hes* negative strains

In recent years, there has been an increasing interest in the study of LEE-negative STEC strains such as O91 because they have been associated with severe disease [5, 27]. The present and previous results have shown that STEC strains can adhere to HEp-2 cells [7, 21]. Some putative adhesins have been characterized to inquire about the adherence mechanisms of STEC strains to epithelial cells [12, 19, 28]. In addition, *hes* was detected in 40% and 46% of LEE-negative STEC strains isolated from humans and cattle [8, 9]. Therefore, to evaluate whether the *hes* gene can confer colonization-associated phenotypes, O91 strains and mutant strains were evaluated. We observed that the O91 strains could adhere to HEp-2 cells, whereas the *E. coli* HB101 strain, used as a negative control, could not (Fig. 1). In the O91Δ*hes* STEC strain and in the *hes* negative-STEC strains, the capability to adhere to HEp-2 cell was variable. However, a non-significant difference was observed between STEC strains, whether positive to *hes* (LAA complete) or negative to *hes* (LAA incomplete) (p = > 0.05). Many different genes mediate the adherence between STEC and host cells and their expression could be affected in a complex way by physiological and environmental factors [29]. Even more, it has been established that STEC strains present varied adherence, different gene expression levels, and alternative genes performing similar functions in host cells. *E coli* HB101pVB1_*hes* showed an increase in the number of adherent bacteria compared with *E. coli* HB101 (p = < 0.05) (Table 1; Fig. 1). These results indicate that Hes is a functional protein in agreement with Montero, et al. [8] and could participate in the adherence to epithelial cells.

Since LAA-positive STEC strains carrying the *hes*, *iha*, and *tpsA* genes showed an adherence capability, we performed a quantitative real-time PCR on the total RNA extracted from the adhered and non-adhered bacteria to HEp-2 cells. Expression levels of each strain were expressed as fold change values relative to their non-adhered strain (Table 3, Figure S5 to S16). The amplification efficiencies of each gene were 98% (*hes*), 91.4% (*iha*), 92.03% (*tpsA*), and 99.6% (*tufA*) (Figure S1, S2, S3, S4).

**Table 3:** Strain ID and fold change

Strain ID	Fold Chang*e hes*	Fold Change *iha*	Fold change *tpsA*
AP16-1	10.90	19.05	68.15
TRN 5-1-1	0.36	1.97	1.02
VO7-4-4	0.70	0.58	0.38
*E. coli* HB101 pVB1_*hes*	1.60	-	-

According to the expression analysis of the *hes* gene, heterogeneous expression levels were detected among the adhered strains studied. In VO 7-4-4 and TRN 5.1.1 strains, the expression levels were lower than in the non-adhered ones, while the expression levels of AP 16.1 strains were higher than those in non-adhered strains. The expression levels of *E. coli* HB101pVB1_*hes* were higher than in the non-adhered strains, detecting a 1.6-fold increase in *hes* expression. Our study is the first to evaluate the expression of *hes* in O91 strains, suggesting that *hes* could participate in the adherence to epithelial cells. Moreover, Montero, et al. [8] demonstrated that sera from patients with HUS are reactive to *hes*, suggesting that this gene participates in human infection.

**Fig. 2:**
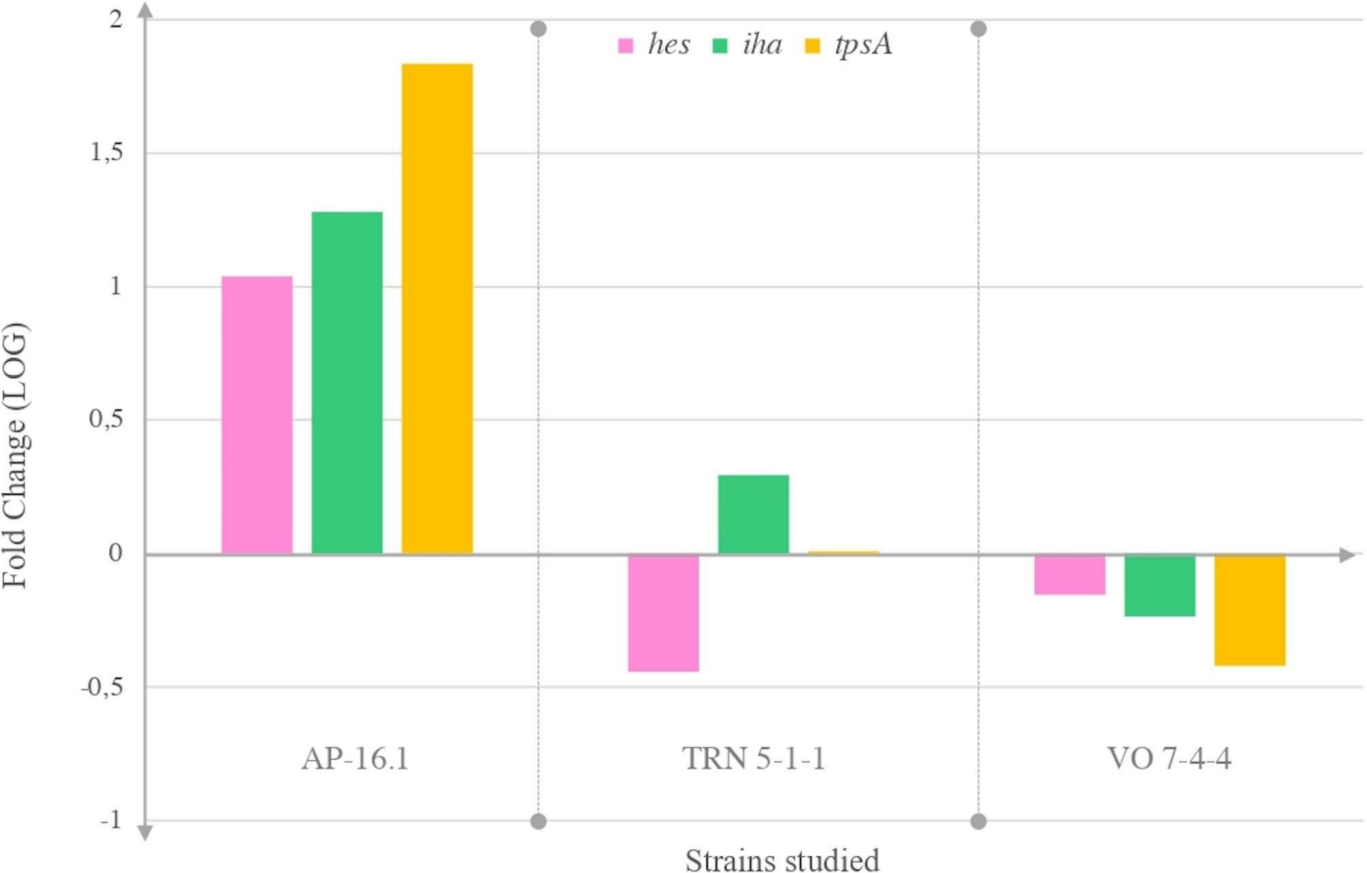
Relative expression levels of STEC strains Relative expression levels of *hes*, *iha*, and *tpsA* in adherent O91 STEC strains. Data are expressed as log 10-fold change values relative to the non-adhered strains. Numbers on the bars indicate each fold change for each gene

For *iha* gene, the VO 7-4-4 strain showed lower expression levels than the non-adhered strains. The expression levels of TRN 5.1.1 and AP 16.1 strains were higher than in the non-adhered strains (Table 3; Fig. 2). Some researchers have studied the mechanisms and functions of *iha* expression in STEC O157 in different assays [30, 31]. However, in a previous study, the alignment and phylogenetic analysis revealed that *iha* LAA had a lower sequence similarity regarding *iha* gene in STEC O157. These results suggest that *iha* genes from LEE-negative and LEE-positive STEC strains may have different origins. In addition, many STEC carries two or more copies of *iha* [10], but we did not know whether *iha* LAA are functional. For this reason, we made an expression assay of *iha* LAA to observe whether it confers adherence phenotype, whose results suggested that Iha LAA acts as an adhesin.

Results for *tpsA* were shown to be the same as for *iha* genes. The *tpsA* has different functions that could participate in the interactions of the bacteria with their host tissue. To colonize their animal hosts, STEC produces several adhesive structures and proteins, many of which are important virulence factors [16]. These adhesins have highly diverse structures, which implies that they physically interfere with each other by the presence of one surface structure obstructing the activity of another [14].

LAA was the most prevalent PAI among LEE-negative STEC strains, suggesting it plays an important role in pathogenesis [7, 9]. Genes encoded in LAA contribute to the adhesion phenotype, but it is not sufficient on their own to govern the adhesion of cells, and at least another factor show signs of cooperating in the adhesion. The expression of STEC adhesins is a coordinated event depending on the strain and its environment as well as its genetic background, a phenomenon named phenotypic plasticity [32].

### Limitation

The main limitation of this study was the differences between STEC strains in the adherence to cells and the expression of genes encoded in LAA, because the strains vary in their genetic background, which allows them, in the absence of some genes, to replace their functions with others. Therefore, this result could be affected in a complex way by physiological and environmental factors. Further research is needed to understand better the mechanism of adhesion and simultaneous expression of genes in the pathogenicity of LEE-negative STEC, a subgroup responsible for illnesses in humans.

### Electronic supplementary material

Below is the link to the electronic supplementary material.

Supplementary Material 1

## Data Availability

All data generated or analyzed during this study are included in this published article.

## Abbreviations

STEC: Shiga toxin-producing *Escherichia coli*
LEE: Locus of Enterocyte Effacement
A/E: Attaching and effacing
PAI: Pathogenicity island
LAA: Locus of Adhesion and Autoaggregation
CT: Threshold cycle
