# Supplementary material for: Expression of hes, iha, and tpsA codified in locus of adhesion and autoaggregation and their involvement in the capability of shiga toxin-producing Escherichia coli strains to adhere to epithelial cells
Source: BMC Res Notes. 2023 Aug 7;16:163. doi: 10.1186/s13104-023-06433-9 (PMC10408066; doi:10.1186/s13104-023-06433-9)
Supplement: Supplementary file 1 — Supplementary Material 1 [file 13104_2023_6433_MOESM1_ESM.docx]

**Figure S1: Standard Curve *tufA* gene.**


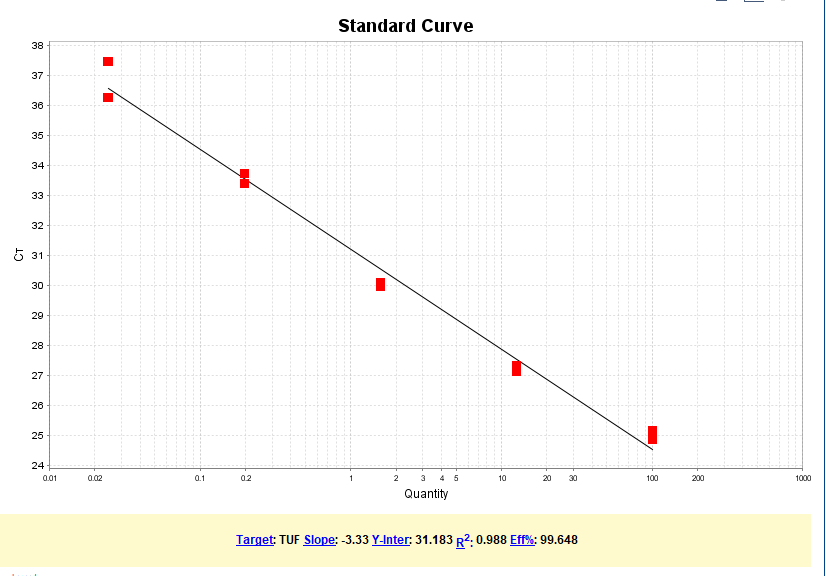


Standard curve and amplification plot of the housekeeping gene *tufA* generated by qPCR amplification of serially diluted purified cDNA of *E. coli* HB101pvb1*_hes* log of genome equivalents/reaction. The trend line equation and the corresponding square regression coefficient (𝑅2) are shown.

**Figure S2: Standard Curve *hes* gene.**


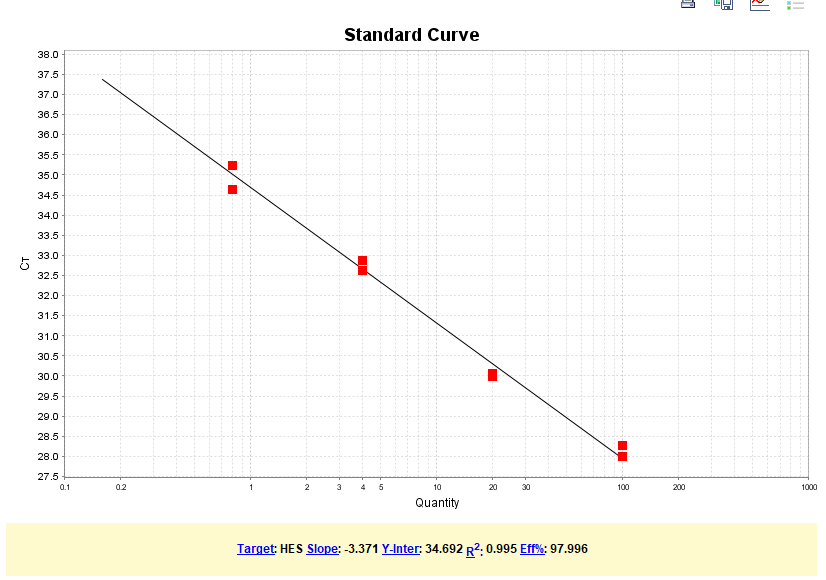


Standard curve and amplification plot of the 113 bp *hes* gene fragment generated by qPCR amplification of serially diluted purified cDNA of *E. coli* HB101pvb1*_hes* log of genome equivalents/reaction. The trend line equation and the corresponding square regression coefficient (𝑅2) are shown.

**Figure S3: Standard Curve *iha* gene.**


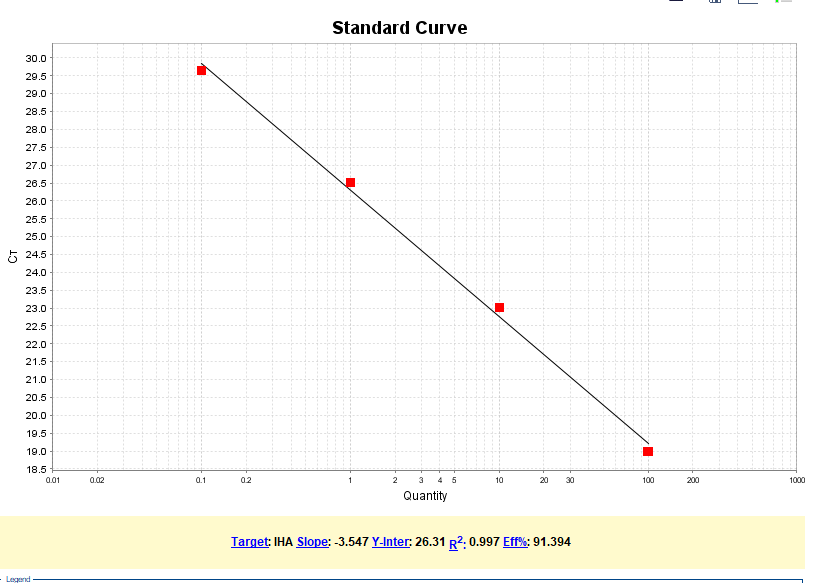


Standard curve and amplification plot of the 172 bp *iha* gene fragment generated by qPCR amplification of serially diluted purified cDNA. The trend line equation and the correspondings square regression coefficient (𝑅2) are shown.

**Figure S4: Standard Curve *tpsA* gene.**


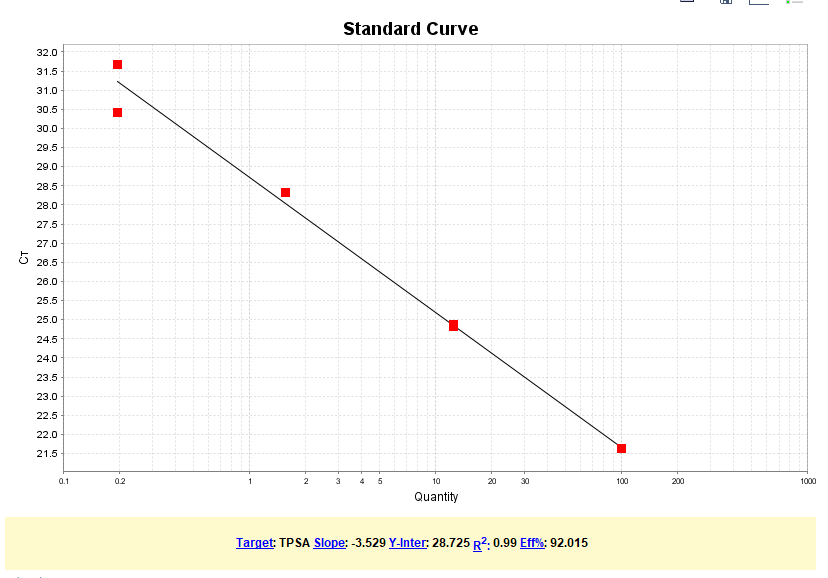


Standard curve and amplification plot of the 174 bp *tpsA* gene fragment generated by qPCR amplification of serially diluted purified cDNA. The trend line equation and the corresponding square regression coefficient (𝑅2) are shown.

**Figure S5: Amplification of samples in logarithmic scale of *tuf* gene expression.**


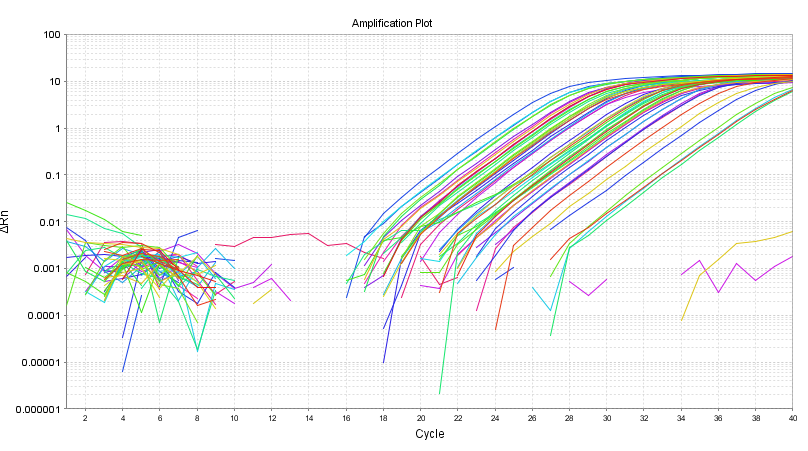


**Figure S6: Amplification of samples in linear scale of *tufA gene* expression.**


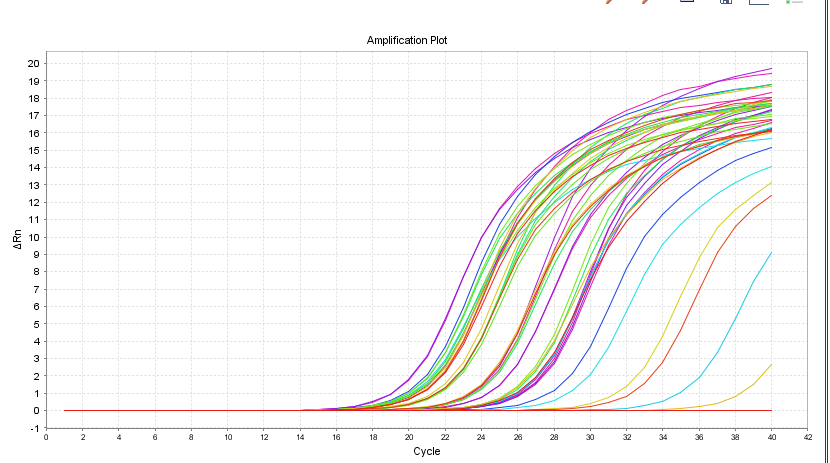


**Figure S7: Amplification the melting curve of samples of *tuf gene* expression.**


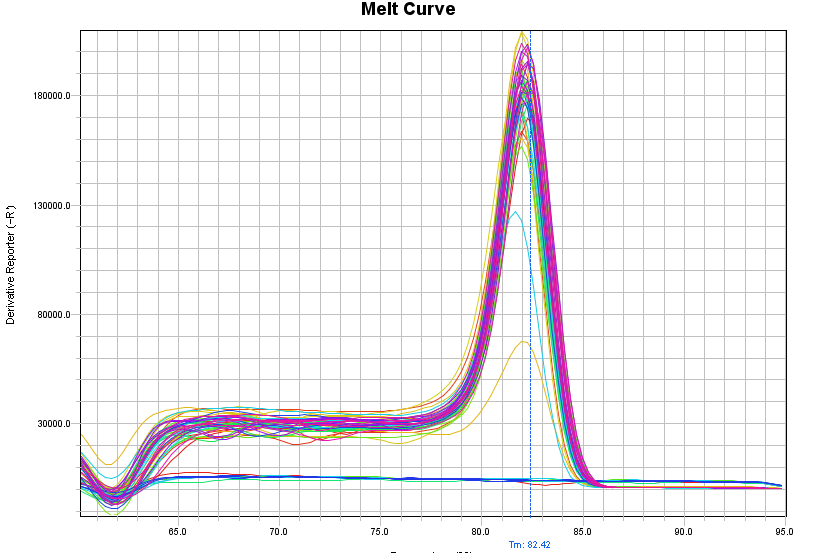


*tufA*: Amplification curves corresponding to amplification of the *tuf* gene by qPCR on a logarithmic scale (Figure S14) and linear scale (Figure S15). Dissociation curves of the qPCR amplification product with an average dissociation temperature of 82.42 ºC (Figure S16).

**Figure S8: Amplification of samples in logarithmic scale of *hes* gene expression.**


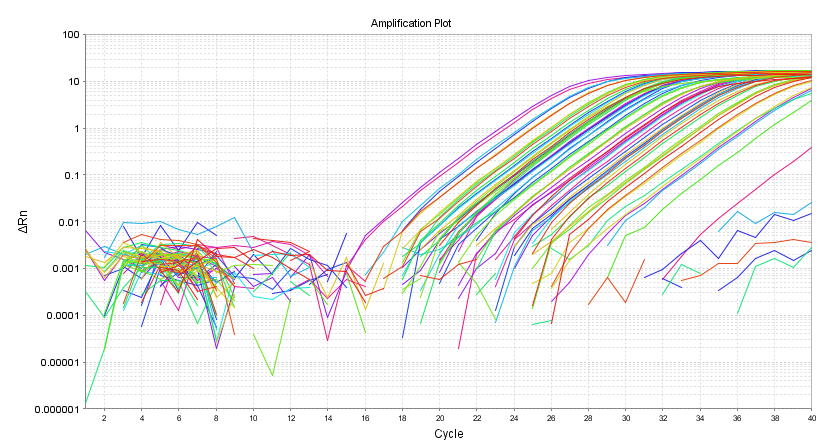


**Figure S9: Amplification of samples in linear scale of *hes gene* expression.**


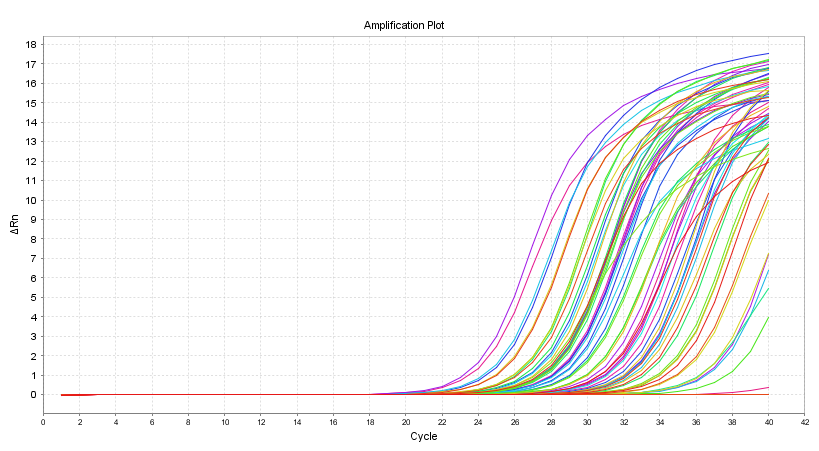


**Figure S10: Amplification the melting curve of samples of *hes gene* expression.**


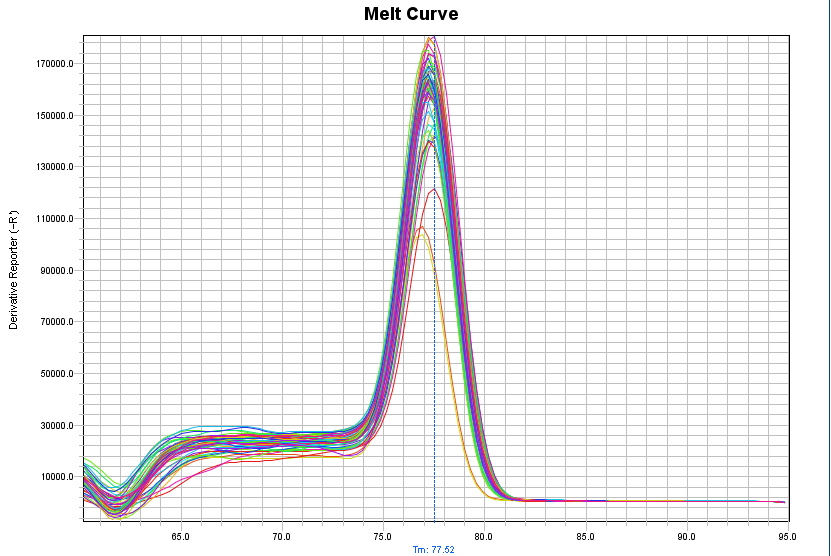


*hes*: Amplification curves corresponding to amplification of the *hes* gene by qPCR on a logarithmic scale (Figure S11) and linear scale (Figure S12). Dissociation curves of the qPCR amplification product with an average dissociation temperature of 77.5 ºC (Figure S13).

**Figure 11: Amplification of samples in logarithmic scale of *iha* gene expression.**


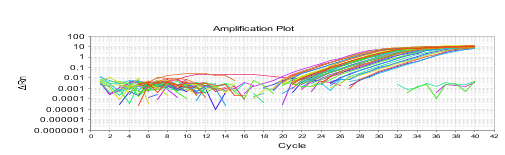


**Figure S12: Amplification of samples in linear scale of *iha* gene expression.**


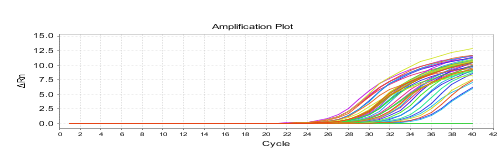


**Figure S13: Amplification the melting curve of samples of *iha gene* expression.**


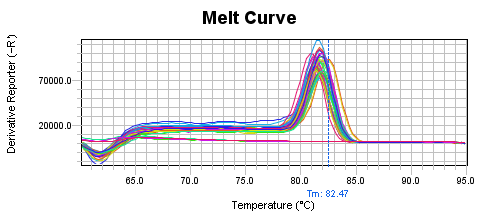


*iha*: Amplification curves corresponding to amplification of the *iha* gene by qPCR on a logarithmic scale (Figure S8) and linear scale (Figure S9). Dissociation curves of the qPCR amplification product with an average dissociation temperature of 82.47ºC (Figure S10).

**Figure S14: Amplification of samples in logarithmic scale of *tpsA gene* expression.**


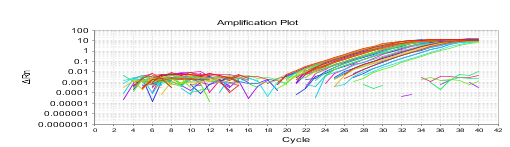


**Figure S15: Amplification of samples in linear scale of *tpsA gene* expression.**


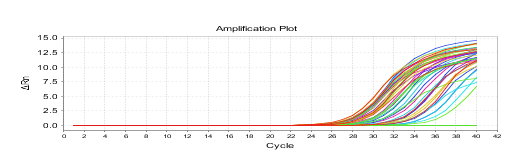


**Figure S16: Amplification the melting curve of samples of *tpsA gene* expression.**


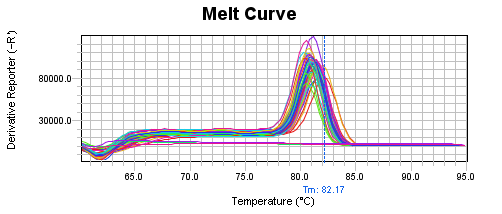


*tpsA*: Amplification curves corresponding to amplification of the *tpsA* gene by qPCR on a logarithmic scale (Figure S5) and linear scale (Figure S6). Dissociation curves of the qPCR amplification product with an average dissociation temperature of 82.17 °C (Figure S7).
